# Supplementary material for: Are carbon emissions trading and green financial instruments synergistic? -Comprehensive quantitative research based on content analysis
Source: PLoS One. 2024 Mar 7;19(3):e0298601. doi: 10.1371/journal.pone.0298601 (PMC10919601; doi:10.1371/journal.pone.0298601)
Supplement: S1 Data — (ZIP) [file pone.0298601.s001.zip › Data/地级市Z_F工作报告.zip!/║╙─╧/σo╤⌠╩╨/└┤╫╘╣1⁄2╓┌║┼í╛┬φ┐╦╩2╛▌═°í┐_2014.docx]

各位代表：

现在，我代表市人民政府，向大会报告政府工作，请予审议，并请各位政协委员提出意见。

一、过去五年工作回顾

2009年以来的五年，是濮阳发展历程中具有里程碑意义的五年。在省委、省政府和市委的正确领导下，我们认真贯彻落实党的十七大、十八大精神，励精图治，拼搏进取，开启了濮阳快速发展的新阶段。五年来，特别是近三年多来，我们深入开展“一创双优”活动，创新观念，振奋精神，创造了强大的人和优势，形成了激情迸发创大业、众志成城图赶超的生动局面。五年来，我们按照市六届人大会议确定的目标任务，紧紧围绕建设富裕和谐美丽新濮阳的宏伟目标，致力科学发展、赶超发展，深入实施“4321”发展战略，坚持“1234”工作思路，积极应对复杂的宏观经济形势，综合施策，攻坚克难，实现了经济发展逆势上扬，成功迈出了“二三五”赶超目标的关键两步，圆满完成了市人大会议确定的目标任务。五年来，濮阳大地发生了历史性变化，取得了令人瞩目的成就。当前的濮阳，发展已驶入快车道，站到了新的历史起点上。

（一）经济发展蒸蒸日上

2013年，全市生产总值突破千亿元大关，完成1130.48亿元，是2008年的1.7倍；规模以上工业增加值643.74亿元，是2008年的1.9倍；固定资产投资941.89亿元，是2008年的3.4倍；公共财政预算收入60.51亿元，是2008年的2.4倍；社会消费品零售总额365.34亿元，是2008年的2.2倍；城镇居民人均可支配收入21571元，是2008年的1.7倍；农民人均纯收入7904元，是2008年的1.9倍；万元生产总值能耗五年累计下降16.82%。

特别是从2011年开始，各项工作全面发力，各项经济和社会发展指标增长速度大幅提升。生产总值增速2010年居全省第15位，2011年上升到第11位，2012年跃居全省第3位，2013年晋升至第2位，三年提升了13个位次。2013年，规模以上工业增加值增速跃居第5位，较2008年上升13个位次；公共财政预算收入增速跃居第4位，上升12个位次；固定资产投资增速跃居第2位，上升12个位次。连续三年荣获“河南省政府目标管理优秀单位”。

（二）产业实力显著增强

工业经济快速发展。深入实施“工业强市”战略，坚持顶层设计，工业规模迅速扩张，质量效益明显提高。至2013年，全市规模以上工业企业达到846家，实现主营业务收入2581.02亿元，税收110.69亿元，利润220.45亿元，较2008年分别增长45%、123%、72%、97%。实施了中原乙烯甲醇制烯烃、丰利石化、龙润化工、联鑫化工等一批重点项目，谋划推进了中原炼厂扩能改造、中原乙烯扩能、河南能化气化岛、岩盐资源开发等一批重大项目，以石油化工为龙头，以煤化工、盐化工为两翼，以精细化工、化工新材料、高端化工制品为发展方向的“三化”融合链接示范基地雏形显现；信宇机械、南方家私、萧山羽绒产业园等一批项目的建设，加快了石油机械装备制造、家具制造、羽绒制品及服饰加工等传统优势产业的集聚集群发展；天能循环经济产业园、绿能高科、惠成电子等项目的实施，培育了新能源、新材料、节能环保等战略性新兴产业。产业集聚区从无到有、蓬勃发展，路、水、电、气、讯等基础设施不断完善，污水处理实现全覆盖，公共服务功能不断增强。累计入驻企业1199家，完成投资1267.92亿元。2013年，产业集聚区规模以上工业增加值达到184.32亿元，较2009年建区之初增长143%。

服务业发展势头强劲。银座、丹尼斯、亿丰等一批知名商贸企业进驻濮阳，濮阳路港国际物流园、开州城市综合体等一批重大商贸物流项目加快建设，商务中心区和特色商业区“两区”建设扎实推进，商贸物流业得到快速发展。城市信用社成功改制为濮阳银行，村镇银行建设步伐加快，金融服务体系不断完善。2013年各项贷款余额381.71亿元，是2008年的2.4倍；各项存款余额突破千亿元大关，达到1012.48亿元。10家企业实现股权交易，成功发行两期城投债，与大型企业集团的战略合作得到加强，资本市场融资工作实现新突破。濮阳国际杂技文化产业园等项目顺利实施，文化旅游产业得到进一步发展。2013年，服务业完成投资195.63亿元，是2008年的4倍。

农业基础更加牢固。实施了高标准粮田“百千万”工程，粮食年产保持在50亿斤以上，实现了十连增。农业产业化迈出新步伐，4个集群被列为省级农业产业化集群，新增市级以上龙头企业89家。新建高标准规模养殖场150个。农业农村五项重点工作成效显著，流转土地77万亩，新建现代农业示范园区155个；开展土地整理，新增耕地6.38万亩；植树8000余万株；实施了金堤河二期治理、大中型灌区续建配套等一批农田水利项目。启动实施了黄河滩区扶贫开发五年攻坚行动。2013年，农业增加值完成148.62亿元，较2008年增长24.5%。

（三）发展优势持续积累

创造了区位新优势。晋豫鲁铁路濮阳段主体完工，郑濮济高速铁路纳入国家铁路网规划；濮范高速、大广高速濮阳段建成通车，德商高速范县段、南林高速东延加快建设，范台梁高速开工在即，国道、省道升级改造工程全面实施。五年来，新增高速公路82.4公里，新改建干线公路360公里，新建改造农村公路2381公里。综合交通体系不断完善，濮阳的区位特点正在转化为区位优势。

汇聚了资源要素。文96天然气调峰储气库建成投用，文23战略储气库获得国家“路条”，中石化中原天然气有限公司落户濮阳并投入运营，谋划实施了一批重大输油输气管网工程，积极推进煤炭储运中心建设，濮阳作为综合能源物流中心的地位日益凸显；岩盐、煤炭资源加快勘探开发，龙丰热电2×60万千瓦机组开工奠基，濮东500千伏输变电工程加快建设，南水北调中线配套工程主体完工，引黄入冀补淀工程顺利推进，濮阳形成了资源能源新优势。

赢得了政策支持。濮范台扶贫开发综合试验区列入《中原经济区建设规划》，实施方案获省批复，得到上级的大力支持；黄河滩区扶贫搬迁纳入省“三山一滩”重点脱贫工程；中石油进一步扩大了产业扶贫范围。濮范台扶贫开发综合试验区已成为濮阳的“金字招牌”。濮阳被列为国家资源枯竭型转型试点城市，累计获得国家支持转型发展资金8.2亿元。开发区晋升为国家级经济技术开发区，并成功创建为国家循环化改造示范试点园区。我市被列入全国第一批新能源示范城市创建名录。一系列的积极运作，濮阳形成了政策新优势。

增强了发展后劲。几年来，谋划实施了一大批事关濮阳未来发展的重大项目。2013年，98个项目列入省重点，是2008年的6倍；完成投资336.13亿元，是2008年的15倍，项目数量和投资额分别位居全省第4位和第3位。五年来，共谋划实施928个省市重点项目，完成投资1605.71亿元。其中，列入省重点项目183个。重点项目的持续加力，筑牢了基础，壮大了产业，濮阳形成了发展新优势。

（四）城乡面貌日新月异

市域中心城市建设取得重大突破。120平方公里的濮阳市城乡一体化示范区获省批复，45平方公里起步区建设掀起高潮，核心区路网框架基本形成，水库有望在三季度蓄水，教育园区加快建设，医专新校区基本建成，市工人文化宫、市图书馆新馆等一批项目扎实推进，示范区正在成为展示濮阳形象的新名片。建成区综合提升大见成效，基本完成了城市路网改造工程，新建改建道路96条、180多公里。实施“以拆促建”，25个城中村、3大旧城片区、5个集贸市场改造扎实推进。全面启动了城市绿系、水系和亮化美化工程建设，新增绿地面积396万平方米，城区河道治理取得阶段性成效，城市夜景和主干道景观亮点纷呈。地下水源给水工程、第二污水处理厂、生活垃圾无害化处理厂等建成投用，城市基础设施得到加强。数字化城管系统投入运行，城市精细化管理水平大幅提升。濮阳县城、清丰县城、濮东组团的功能进一步完善，与中心城区融合步伐加快，“一中心三组团”的发展格局基本形成，为濮阳建设区域性中心城市打开了广阔空间。

县域中心城市、小城镇和新农村建设取得新成效。强化基础设施建设，不断完善公共服务功能，着力提高管理水平，县域中心城市面貌焕然一新。濮阳县创建为省级文明城，清丰、范县创建为省级文明城、卫生城、园林城。小城镇建设取得新进展，实力不断壮大，活力不断增强，17个乡撤乡建镇，16个乡镇创建成为省级生态乡镇。加快新型城镇化进程，积极促进农村人口有序转移，2013年城镇化率达到36.7%，较2008年提高2.9个百分点。新农村建设扎实推进，农村生产生活条件进一步改善，村容村貌明显改观。

（五）动力活力竞相迸发

重点领域改革迈出新步伐。加快转变政府职能，深化行政审批制度改革，压减下放审批事项152项，清理取消前置条件89项，优化了程序，提高了效率。创新体制机制，制定出台了谋划、决策、落实、督导、总结等一整套工作制度，提高了决策水平和执行能力。创新政府投资项目管理机制，强化监管，节约了资金，提高了效益。推进国有企业改革，完成了市直部门所属企业脱钩改制，国有资本运营平台建设取得新成效。扎实推进事业单位分类改革、医药卫生体制改革、财政管理体制改革，文化、教育、科技等领域的改革都取得了新进展。

民营经济迅速发展。强化政策支持，优化发展环境，民营经济不断发展壮大。2013年民营经济完成增加值729.5亿元，占全市GDP的64.5%，较2008年提高11.4个百分点。濮耐集团荣获“2013年度省长质量奖”。“雪鸟”羽绒、“登星”家具荣获“中国驰名商标”。蔚林股份等9家企业荣获“河南省国际知名品牌”。

对外开放进一步扩大。招商引资成效显著，成功引进了天能集团、龙润化工、南方家私、全友家私、亿丰时代广场、荣事达等一批带动能力强、科技含量高的重大产业项目和知名企业。五年来，累计落地亿元以上项目689个，利用省外资金458.9亿元，年均增长30.3%；累计到位境外资金10.3亿美元，年均增长50.9%。专用化工品、羽绒服装及制品两个基地被确定为国家外贸转型升级示范基地，电光源、石油装备等4个基地被确定为河南省出口基地。

创新驱动能力不断增强。我市连续两届被评为“全国科技进步先进市”。市农业科技园区成功创建为国家级农业科技园区。五年来，科技投入逐年增加，新增高新技术企业24家，新建省级工程技术研究中心11家、市级工程技术研究中心34家、重点实验室9家，建成了省级科技企业孵化器，在孵企业25家。国家电光源质检中心主体完工。新增国家级知识产权优势企业2家，专利工作保持全省先进位次。

油地战略合作不断深化。油地携手并进、共兴共荣的“三种意识”深入人心，支持油田发展的“三个好环境”蔚然形成，油地战略合作结出丰硕成果。建立了油地高层联席会议制度，油地双方同心协力，促成了中石化中原天然气有限公司入驻濮阳，谋划推进了中原炼厂扩能改造、地热资源开发、盐矿资源综合利用、高端石油机械装备制造研发中心建设等一批重大项目，实施了油田改制企业“退城入园”、棚户区改造、城区河道治理、道路改造、供热管网配套等一批重点工程。油地呈现出互促共进、共建美好家园的良好局面，成为全国油地合作的典范。

（六）民生保障水平明显提高

五年来，全市财政民生支出474.9亿元，占比位居全省前列。每年着力抓好十项重点民生工程，切实解决涉及人民群众切身利益的实际问题。就业和社会保障水平不断提高，新增就业44.6万人，城乡居民社会养老保险制度实现全覆盖，企业退休人员基本养老金逐年增长，新农合、城镇居民医疗财政补助标准提高到280元，城乡低保对象月人均保障标准分别达到300元、150元，农村五保对象集中、分散年供养标准分别达到3200元、2220元。保障性住房开工建设63421套，竣工15344套。农村生活条件不断改善，提前两年解决了非滩区180万农村人口的安全饮水问题，走在了全省前列。改造农村危房25600套。各项社会事业全面发展，实施中小学校安工程项目641个，新建校舍107万平方米，消除中小学危房73.91万平方米，建成标准化学校371所。圆满完成了学前教育三年行动计划，新建扩建幼儿园412所。基础教育和高中教育质量明显提高，职业教育长足发展，高等教育取得新突破。加强医疗和公共卫生服务体系建设，新建扩建市县医院等医疗卫生机构23个，新增床位2600张，启动了乡村医疗机构标准化建设攻坚行动。人口自然增长率控制在6.5‰以下。文化事业繁荣发展，实施了广播电视村村通、农家书屋等一批文化工程。豪艺杂技集团命名为河南省杂技集团，《水秀》荣获中国杂技最高奖“金菊奖”优秀剧目奖，《大红灯笼》剧本荣获第20届曹禺戏剧文学奖。群众体育蓬勃开展，竞技体育成绩显著。实施公交体制改革，新购环保公交车119辆，改善了市民出行条件。实施了食品安全“四大放心工程”，连续三年荣获“全省食品安全工作优秀市”称号。扎实做好依法治市、信访稳定、安全生产、社会治理等工作，群众安全感和执法满意度调查均居全省第一。

（七）政府自身建设不断加强

坚持依法行政，法治政府建设迈出新步伐。认真执行人大及其常委会决议和决定，自觉接受监督，支持政协参政议政，切实做好人大代表建议和政协委员提案办理工作，广泛听取民主党派、工商联、无党派人士意见，支持工会、共青团、妇联等人民团体开展工作。深入开展“一创双优”活动，扎实推进“两转两提”，深化政务公开，公务员素质和行政效能不断提高。加强廉政建设，落实“一岗双责”，认真落实中央八项规定精神和省、市委有关要求，严格控制行政经费和一般性支出，切实加强行政监察和审计监督，树立了清正廉洁的良好形象。同时，民族宗教、统计、发展研究、外事侨务、机关事务、残疾人工作、气象、地震、人防、史志、档案等各项工作都取得了新成绩。大力开展国防教育，扎实做好双拥工作。驻濮解放军和武警部队在支持地方发展、维护社会稳定方面做出了积极贡献。

各位代表！过去的五年，是濮阳负重爬坡、奋力赶超的五年，是值得铭记、令人自豪的五年。五年的艰辛探索，五年的励精图治，五年的矢志奉献，开创了大建设、大发展、大跨越的崭新局面，谱写了加快建设富裕和谐美丽新濮阳的壮丽篇章。濮阳大地生机勃发，大事要事捷报频传，赶超发展硕果累累，濮阳人民更加自信！这些成绩是省委、省政府和市委正确领导的结果，是市人大、市政协大力支持的结果，是全市人民凝心聚力、拼搏进取的结果。在此，我代表市政府，向工作在各条战线的工人、农民、知识分子、干部、人民解放军指战员、武警官兵、公安民警以及关心、支持濮阳发展的各界人士致以崇高的敬意，表示衷心的感谢！

各位代表！五年的团结奋斗，深化了我们对赶超发展的认识，获得了宝贵的经验。主要有：

——必须坚持深入开展“一创双优”活动。“一创双优”活动开启了濮阳赶超发展的新阶段。观念引领发展，观念的创新，破除了禁锢，激活了思想，打开了广阔的发展空间；作风推动发展，作风的优化，锻造了求真务实、拼搏进取的干部队伍，干出了非凡的业绩；环境保障发展，环境的优化，创造了发展新优势，提升了区域竞争力。“一创双优”活动已成为推动濮阳赶超发展的不竭动力，形成了强大的人和优势，必须坚定不移地持续深入开展。

——必须坚持科学发展赶超发展。实践证明，科学发展、赶超发展的战略决策，符合发展规律、切合濮阳实际、顺应群众期盼，我们咬定发展不放松，闯出了一条欠发达地区跨越发展的新路子，赢得了广泛赞誉。面对全面建成小康社会的艰巨任务，面对日趋激烈的区域竞争，我们必须继续高扬科学发展、赶超发展主旋律，不动摇、不懈怠，心无旁骛，不断把建设富裕和谐美丽新濮阳的宏伟事业推向前进。

——必须坚持为民利民改善民生。全心全意为人民服务，是党的根本宗旨，是政府的根本责任。任何时候都必须坚持人民至上的执政理念，切实解决人民群众最关心、最直接、最现实的利益问题，让人民群众得到更多的实惠，生活得更加幸福美好。

——必须坚持早干快干多干。早干快干多干是我们加快发展的宝贵经验。只有早干，才能抢占先机；只有快干，才能赢得主动；只有多干，才能实现赶超。正是靠着这种精气神，我们克服了一个又一个困难，办成了一件又一件大事，创造了一个又一个奇迹。赶超发展任务艰巨，不进则退，时不我待，我们必须只争朝夕，大干快上。

——必须坚持以项目为抓手。抓发展归根到底是抓项目。以项目为抓手，各项工作项目化，各个项目台账化，是落实责任、推动工作、取得成效的重要方法。我们必须牢牢抓住项目建设这个“牛鼻子”，以项目的扎实推进，实现经济社会的全面发展。

——必须坚持科学有效的工作机制。机制是做好工作的保障。在赶超发展的实践中，我们探索建立了领导分包、台账管理、重点项目周调度、观摩考评等制度，构建了推动工作的新机制，激发了干劲，提高了执行力，形成了明争暗赛、竞相发展的生动局面。这套机制好使管用，必须不断总结完善，认真执行。经验弥足珍贵，我们必须倍加珍惜，发扬光大，以创造无愧于时代、无愧于人民的辉煌业绩！

在肯定成绩的同时，我们清醒地认识到，我市经济社会发展仍面临一些困难和问题：一是基础差、底子薄，经济总量不大、人均水平较低，仍是制约我市赶超发展的主要因素。二是产业发展中的深层次矛盾依然突出，工业发展集群化水平不高，自主创新能力不强，高新技术企业发展不快，调整结构、转型发展任务艰巨；现代服务业发展滞后；农业增效、农民增收难度加大。三是支撑发展的基础尚不稳固，基础设施配套有待完善，聚要素、创优势的能力亟待增强；支撑发展的大项目、好项目少，招商引资需进一步加力。四是改革力度不够，开放水平不高，民营企业数量少、规模小，人才支撑能力不强，改革开放需进一步深化。五是城镇化率仍低于全国、全省平均水平，吸纳就业能力和承载产业能力有待提高。六是社会治理还有不少薄弱环节，改善民生任务繁重，生态文明建设需进一步加强。七是政府职能转变还不到位，思想观念、工作作风、发展环境等方面仍存在一些突出问题。对此，我们一定高度重视，认真解决。

二、今后五年目标任务

今后五年，是贯彻落实党的十八大精神、全面建成小康社会的关键时期，是我市爬坡过坎、实现赶超的决定性阶段。我们要紧紧围绕中央、省和市委的决策部署，认真落实市七届人大会议的各项决议，以“一创双优”活动为动力，牢牢把握建设石油化工、煤化工、盐化工“三化”融合链接示范区、中原经济区重要出海通道、中原经济区与环渤海经济圈衔接融合的前沿、省际交会区域性中心城市的战略定位，抢抓发展机遇，深化改革开放，强化创新驱动，加大基础设施建设力度，加快新型工业化、新型城镇化步伐，不断夯实农业基础，统筹城乡发展，协调推进各项社会事业，着力改善民生，推动科学发展、赶超发展，加快建设富裕和谐美丽新濮阳。今后五年的主要目标是：

——经济实力大幅跃升。到2018年，生产总值、公共财政预算收入实现“两个翻番”。生产总值突破2000亿元；公共财政预算收入超过120亿元；全社会固定资产投资年增速保持在20%以上，经济基础更加坚实。

——产业发展实现转型升级。大力实施工业发展“1062”工程：到2018年培育10家以上年主营业务收入超100亿元的大型企业；6个以上年主营业务收入超300亿元的产业集群；2个以上年主营业务收入超1000亿元的产业。新能源、新材料、节能环保、生物医药等战略性新兴产业实现新突破，成为新的经济增长点。初步形成特色鲜明、质量效益高、竞争力强的现代工业体系。第三产业快速发展，大商贸、大物流、大文化、大旅游的发展格局初步形成。农业生产条件明显改善，粮食年产稳定在50亿斤以上，新型农业经营体系初步形成，现代农业产业化集群规模不断壮大，实现农业增效、农民增收。

——区域性中心城市地位初显。综合交通体系框架形成，范台梁高速建成投用，郑濮济高速铁路、濮阳至阳新高速开工建设，濮阳成为名符其实的中原经济区重要出海通道，区位优势充分彰显；总部经济加快发展，资源要素加速汇聚，区域经济互动增强；城市形态更加完备，城乡一体化示范区基本建成。现代服务业发展壮大，打造区域性高端医疗服务中心、教育培训中心、休闲度假中心，城市首位度得到显著提高。

——城乡协调发展格局基本形成。城镇化率达到50%左右，形成以市域中心城市为引领、以县域中心城市为承接、以小城镇为支点的新型城镇体系，产业支撑力和人口承载力大幅提升，加快基础设施和公共服务向农村延伸，扎实推进新农村建设，实现城乡繁荣、人民幸福安康。

——改革开放进一步深化。重点领域改革取得突破，体制机制不断完善，发展环境明显改善，各类市场主体更具活力，人力资源优势进一步发挥，创新能力显著提升，对外开放深度和广度进一步扩大，政府公信力和公共服务能力全面增强。

——人民群众生活水平明显提高。城镇居民人均可支配收入、农民人均纯收入年均增长10%以上；城乡差距进一步缩小；年新增就业7万人以上。基本社会保障、基本医疗卫生服务实现全覆盖，住房保障体系得到完善，全民受教育程度和人才支撑能力进一步提升，人民群众文化生活更加丰富，社会更加和谐稳定。

——生态文明建设取得新成效。全社会节约意识、环保意识、生态意识明显增强，全面完成省定节能减排目标。完成绿系、水系建设，完善生态保护体系；环保基础设施覆盖城乡，形成较为完善的环境防控体系；循环绿色低碳生产模式广泛推广，产业循环体系建设取得突破，形成天蓝、地绿、水清的优美环境。

各位代表，前景辉煌灿烂，目标令人鼓舞。经过五年的不懈努力，我们的目标一定能够实现，濮阳的明天一定会更加美好！

三、2014年重点工作

2014年是全面深化改革的一年，是实现“二三五”赶超目标的关键之年。综合判断宏观经济形势，世界经济持续复苏的基础尚不稳固，我国经济进入中高速发展期，区域竞争进一步加剧，但世界经济复苏态势好于去年，我国经济长期向好的基本面没有改变，全面深化改革、加快城镇化步伐，将释放更多的发展红利；沿海发达地区加快产业转移的趋势没有改变；国家产业政策导向与我市产业发展方向基本契合；我省三大国家战略规划的实施，为濮阳发展提供了机遇。特别是党的群众路线教育实践活动和“一创双优”活动的深入开展，以及经济社会的持续赶超，积累了更多的发展优势，提供了强劲动力。总的来看，面临的有利因素大于不利因素，赶超发展的基本条件没有改变。为此，我们必须保持清醒头脑，牢牢把握发展第一要务，抢抓机遇，应对挑战，推动濮阳科学发展、赶超发展。

今年政府工作的总体要求是：深入贯彻落实党的十八大、十八届二中、三中全会精神，以党的群众路线教育实践活动和“一创双优”活动为动力，坚持“4321”发展战略和“1234”工作思路，坚持科学发展、赶超发展，着力改革创新，激发市场活力；着力扩量提质，壮大经济实力；着力优化经济结构，推进经济转型；着力统筹城乡发展，推进社会事业发展，切实改善民生，保持社会和谐稳定，实现经济社会持续健康较快发展。

今年经济社会发展主要目标是：生产总值增长11%以上；规模以上工业增加值增长16%；公共财政预算收入增长12%以上；固定资产投资增长23%左右；社会消费品零售总额增长15%左右；外贸出口总额增长12%；实际利用境外资金增长15%，实际利用省外资金增长20%；城镇居民人均可支配收入和农民人均纯收入分别增长10%、11%；人口自然增长率控制在6.5‰以下；居民消费价格指数涨幅控制在3.5%以内；节能减排实现省定目标。围绕实现上述目标，着重抓好十个方面工作：

（一）强化基础设施建设

加快城乡交通道路建设。谋划推进实施铁路项目3个、高速公路项目4个、干线公路项目9个、城区道路项目20个、农村道路项目500个，构建内畅外联的综合交通体系。铁路方面，晋豫鲁铁路通道濮阳段年内通车，做好郑濮济高速铁路、濮阳至开封城际铁路等项目前期工作。高速公路方面，完成德商高速范县段、南林高速东延主体工程，开工建设范台梁高速，启动濮阳至阳新高速前期工作。干线公路和农村公路方面，完成国道106、省道101、307、212等路段改造项目，开工建设国道341、省道101等路段改造工程。全年新改造干线公路100公里，新建改建农村公路1000公里。市城区道路方面，完成卫都大道、濮旺路等道路续建，启动高阳大道、张仪路等道路建设，推进跨渠桥梁建设，濮阳市城乡一体化示范区完成“十路十桥”工程。建成区完成胜利西路、大庆路、南海路、站南路等道路改造。解决濮上路、长庆路等铁路涵洞积水问题。

强化资源能源保障能力。推进中原天然气调度中心建设，完善豫北、鲁西、冀西南地区骨干输气管网，开工建设文23战略储气库、丰利石化至日东输油管道支线、成品油豫北输油支线等项目。建成濮东500千伏输变电工程，加快龙丰热电2×60万千瓦机组建设，开工建设万众天然气分布式能源项目、盛润背压式汽轮发电机组项目，增加热源，提高供热能力。推进煤炭储配中心、清丰10万千瓦风电等项目的前期工作。加强同中石化新星石油公司合作，推进地热资源综合利用，努力打造全国地热能源综合开发利用示范区。

完善城镇配套服务功能。建成区，启动城区污雨水管网分离工程和公厕建设。新建7座污雨水提升泵站、5座垃圾中转站。加快中心公园建设。加强农贸市场标准化改造和监管，加快中原市场、劳动市场、金宇市场、天龙市场、油田商贸中心等市场改造进度。完善供暖、供水、供气等配套工程，新增集中供暖面积100万平方米以上。濮阳市城乡一体化示范区，完成高压线路迁改。建成濮阳医专，加快建设市工人文化宫、图书馆新馆，推进市民综合活动中心、科技馆、档案馆、体育场、游泳馆、球类运动综合馆等场馆建设，推进新一高、技师学院、市委党校等项目建设，进一步提升公共服务能力。县（区）、乡（镇），按照现代城市理念，搞好规划，切实加大基础设施建设力度。

抓好水利设施建设。引黄调节水库建成投用，完成城区河道综合治理任务，濮上河建成通水，完成南水北调支线工程和市第三水厂建设，争取开工建设引黄入冀补淀工程，加快推进南乐杏元和范县引黄调蓄工程建设。实施大中型灌区续建配套和节水改造工程。推进黄河标准化堤防建设。

（二）壮大工业经济实力

坚持上项目、育产业、扩规模、促转型，实施省市重点工业项目157个，其中10亿元以上项目39个。概算投资955.5亿元，年内投资497.4亿元。

做大做强石油化工产业。以龙头项目和节点项目建设为重点，推进石油化工产业升级再造，促进“三化”融合链接，向精细化、高端化方向发展。加快推进建设河南能化气化岛、丰利石化、中原乙烯扩能改造、中原炼厂扩能改造等重大龙头项目，实施龙润化工二期、晨旭化工异辛烷、君恒生物长链二元酸、沃森科技超高分子聚乙烯等节点项目，支持惠成电子、蔚林化工、迈奇科技等高成长企业发展壮大，提升行业竞争力。

做强做优传统产业。加大对传统优势产业的改造提升，壮大规模，培育品牌，促进集群发展。推进全友、双虎家具产业园等项目建设，促进工贸一体化发展，打造中部家具之都。推进雪鸟羽绒二期、鹏达羽绒二期等项目建设，育龙头，创品牌，打造羽绒制品及服饰加工产业基地。着力引进汇源集团等一批知名品牌企业，促进食品行业提档升级。推动中油特车、双发实业等企业与大型企业集团开展战略合作，促进中原总机厂“退城入园”扩能发展，推进石油装备研究院建设，提高自主创新能力，打造重要的石油及高端装备制造基地。

培育战略性新兴产业。依托天能循环经济产业园、绿能高科等，推进新型动力能源和新能源汽车基地建设；加快荣事达光电产业园、清华同方光电产业园等重大项目建设，着力引进一批高科技项目，培育壮大光电子产业；注重集群引进，发展节能环保产业；加快生物质能产业园建设，谋划推进生物医药产业发展。提升产业集聚区建设水平。按照“四集一转”的要求，进一步明晰主导产业，促进集群发展。扎实推进产业集聚区内村庄整合，强化基础设施、配套服务平台建设，不断提高承载能力。加快城区基础设施和公共服务向产业集聚区延伸覆盖，促进产城互动。年内完成固定资产投资600亿元，主营业务收入900亿元。

（三）加快新型城镇化进程

坚持以人为本、优化布局、生态文明、传承文化、产城互动、科学有序的原则，按照“13363”空间布局，统筹城乡发展，走出一条符合我市实际的新型城镇化道路。

做大做强市域中心城市。抓好第四轮濮阳市城市总体规划修编，优化空间布局，构建“一中心三组团”的发展格局，努力建设区域性中心城市。加快城乡一体化示范区建设，起步区要以水库、路网、项目建设、配套功能完善、村庄整合迁建为重点，统筹安排、强力推进，路网形成框架，水库九月底实现蓄水，岸线配套设施建设和绿化亮化工程同步实施，龙山绿化全面启动，“六大广场”、“十大建筑”加快推进，形成城市建设的新亮点。同时，按照复合型理念，统筹抓好产业发展、生态环境、都市农业等功能区建设。继续加大建成区综合提升力度，抓好市城区河道治理，实现“通、净、绿、亮”的目标，与水库、濮上河相互衔接，形成“五河贯城、两湖相映”的生态水系。完成迎宾路、黄河路等绿化亮化工程，绿化提升面积120万平方米。加快推进城中村、旧城片区改造，年内全面开工建设戚城村等25个城中村安置房项目,力争赵村等5个村部分村民回迁，启动李家村等3个城中村和行政南小区、昆吾西片区2个片区改造。抓好京开大道等主次干道两侧建筑物的改造提升。以数字化城管系统为依托，提升城市精细化管理水平。加快中心城区与濮阳县城、清丰县城、濮东组团和皇甫片区的融合对接，推进一体化发展，提高城市承载力和辐射力。

加快县域中心城市和小城镇建设。县域中心城市，坚持改造、建设、管理并重，继续加强基础设施建设，持续加大综合提升力度，不断完善城市功能，改善城市面貌，提升城市品位，打造彰显地域特点和文化特质的实力县城、魅力县城，进一步增强承载力和吸引力。开展美丽宜居小城镇建设活动，重点抓好13个试点，以点带面，努力建设风格鲜明、充满活力的小城镇。

统筹推进新农村建设。以县（区）为单元，按照产业、新农村、土地、公共服务和生态规划“五规合一”的要求，编制修订完善新农村建设规划，科学确定村庄布局和规模，配套建设基础设施和公共服务设施。继续抓好干道沿线农村环境综合整治，改善提升农村面貌。重点抓好美丽乡村建设“百村示范行动”，建设环境优美、生活幸福、文明和谐的美好家园。

有序推进农业人口转移。依托产业集聚区和城镇，大力发展工业和第三产业，创造更多的就业岗位，促进农村人口向城镇转移。培训农村劳动力2万人以上。新增农村转移就业3万人。落实中央、省关于户籍改革、社会保障等有关政策，保障进城落户农民土地承包经营权、宅基地用益物权和农民集体经济收益分配权等权益，让农业转移人口“转得出”、“进得来”、“落得住”。

（四）大力发展第三产业

加快发展商贸物流业。推进恒丰中央广场、泽鲁现代商贸中心等重大商贸项目建设，加快濮阳路港国际物流园、豫北商贸物流园等重点物流项目建设，努力打造区域性商贸物流中心。

加快发展金融服务业。积极做好金融机构引进工作。争取1至2家农村信用社改制为商业银行。大力发展农村金融，台前德商村镇银行年内开业，推进清丰、南乐设立村镇银行。做好第三期10亿元城投债发行等融资工作，支持引导企业融资发展，资本市场融资力争突破80亿元。

培育发展新兴服务业。顺应消费需求，积极发展信息服务、电子商务、文化创意、教育培训、科技研发、工业设计、服务外包、物联网等新业态，鼓励发展养老、健康、休闲等服务业。抓住我省整合资金支持现代服务业发展的机遇，谋划包装一批新兴服务业重点项目，争取进入省计划盘子。

加快商务中心区和特色商业区建设。完成各项规划编制。充分发挥市场作用，多渠道筹资、多元化投入，推进基础设施建设。着力引进一批优势企业、优质项目，促进服务业集聚发展。将华龙区特色商业区等基础较好的“两区”，率先打造成产业高集聚、产出高效率、功能高复合、就业高容量的服务业集群。

（五）巩固夯实农业基础地位

提高粮食生产能力。实施高标准粮田“百千万”工程，重点打造10个精品万亩示范方、10个精品千亩示范方、20个精品百亩示范方。改善农业生产条件，加强优良品种繁育和推广，不断提高农业机械装备水平，确保粮食年产稳定在50亿斤以上。

推进农业结构调整。围绕促进农业增效、农民增收，进一步优化种植结构，新建百亩以上现代农业园区5个、五百亩以上林果花木基地30个，新增高效农业1.2万亩；大力发展都市生态农业，在城市周边规划建设一批具有城市“菜篮子”功能的现代化农产品贸易市场，建设一批兼具生态绿化、休闲观光等功能的现代农业园区；加快发展畜牧业，新建改建50个标准化养殖场区；实施农业产业化集群培育工程，加大对林板及家具、羽绒制品及服饰加工、畜牧养殖及加工、粮油加工等产业化集群培育力度，新增销售收入超亿元农业产业化龙头企业2家，建成1至2家国家级农业产业化龙头企业。

抓好濮范台扶贫开发综合试验区建设。力争将黄河滩区纳入国家扶贫攻坚范围，争取中石油进一步加大产业扶贫力度，积极探索农村土地承包经营权、宅基地和房屋产权改革，争取国家和省更多的投入和支持。全力抓好黄河滩区扶贫开发五年攻坚行动，加强道路、电网等生产性基础设施建设；加快土地流转，发展特色高效农业；强化滩区群众就业培训，大力发展劳务经济，增加滩区群众收入。积极推进黄河滩区搬迁扶贫工作。

（六）不断提升开放水平

切实将开放带动作为全局性、综合性战略举措，注重区域互动发展，提升对外开放层次和水平，集聚更多要素，促进区域性中心城市建设。

构建大开放发展格局。立足濮阳区位优势，加强与环渤海经济圈对接，搭建产学研合作平台，主动接受辐射；积极谋划推进“无水港”建设，加快商检分支机构建设，争取设立海关，构筑“大通关”平台，建设出海大通道；加强与郑州航空港经济综合实验区对接，建设大物流节点城市，促进区域经济联动发展。发挥我市国家、省出口基地作用，扩大出口，提高经济外向度。

加大招商引资力度。坚持经常性的“走出去、请进来”活动，围绕主导产业，按照产业图谱和招商图谱，突出招大引强，强化产业链招商、集群招商。加强与行业协会和商会的联系，认真研究产业动向和大型企业的战略布局，选准重点目标，找准对接点，实行领导分包，专人负责，“一把手”上阵，力求在招大引强上取得新突破。年内引进落地投资亿元以上项目80个，其中5亿元以上项目30个、10亿元以上项目20个。

强化战略合作。加强与中石化、中石油、中海油、省投资集团、河南能化等大企业的战略合作，促其在濮扩大投资、布局项目。抢抓改革机遇，着力推进油地战略合作向更高层次、更宽领域迈进，在产业发展、城市建设、民生改善等领域，再共同谋划实施一批重大项目，实现融合发展。不断完善油地协调推进机制，将良好的油地关系体现在发展上、体现在民生上、体现在社会综合治理上，共同建设我们的幸福美好家园。

（七）全面深化改革创新

按照中央和省统一部署，结合我市实际，明确重点，把握方向，推动重要领域和关键环节改革取得新进展。

深化政府机构改革。按照优化政府组织结构、建立科学精干高效行政体制机制的要求，做好对机构设置的调整，进一步理顺部门间的职责关系，统筹推进相关改革，严格控制机构编制，确保机构编制总量和财政供养人员只减不增。年底前完成市县政府机构改革任务。

深化国有企业改革。坚持以运作盘活国有资产为核心，分类施策，采取改制、重组、资产注入国控公司等方法，搞好资本运作，重塑市场主体，提高国有资本运营效益。探索解决“壳公司”等历史遗留问题，积极做好厂办大集体企业改革试点工作。完善国资监管体制机制，规范运作，做好产权登记、产权交易、资产评估、资产处置等工作。

大力发展民营经济。按照非禁即入原则，放宽准入领域，拓宽准入渠道，支持民营企业进入交通、能源、市政设施、社会事业等领域。积极发展混合所有制经济，鼓励民营企业参与国企改革，引导民营企业加强与央企、省企的对接合作。开展涉企收费专项治理，再取消和免征一批行政事业性收费。加快中小企业公共服务平台建设，完善扶持政策，做好企业家培训，建设“百名优秀企业家队伍”。

积极推进农村改革。坚持和完善农村基本经营制度，赋予农民更多财产权利。创新农业生产经营体制机制，培育专业大户、家庭农场、农民合作社等新型农业经营主体，发展多种形式适度规模经营，鼓励和引导工商资本和社会资本进入农业领域。

统筹推进其他各项改革。全面落实营改增等改革政策。实施项目预算和预算绩效管理，发挥政府投资管理办公室作用，提高财政资金使用效益。推行政府购买社会服务。推进工商注册制度便利化。加快事业单位分类、医药卫生体制、文化体制等领域改革。

强化创新驱动。提升科技企业孵化器建设水平，积极创建国家级科技企业孵化器。加快创新主体培育，力争新增高新技术企业5家、河南省创新型示范企业3家，新建3个省级工程技术研究中心、12个市级工程技术研究中心。加强产学研合作，促进科技与金融结合、科技与经济结合，实施一批科技成果转化项目。注重品牌带动，争创全国质量强市示范城市。推进羽绒质检中心、家具质检中心、石油钻采设备及装备质检中心建设。鼓励引导企业进行管理创新和商业模式创新，增强核心竞争力。积极申报国家区域战略性新兴产业集聚发展试点和国家新型工业化产业示范基地。

（八）切实推进文化建设

提升全民文明素质。培育和践行社会主义核心价值观，大力弘扬“三李”精神和“阳光、真诚、简单、责任、激情”的濮阳干部文化，积极推进公民道德建设。深入开展学雷锋志愿服务活动等群众性精神文明创建活动，做文明人、办文明事，在全社会形成勤劳节俭、尊德守礼、孝老敬老、读书学习的良好风尚。汲取传统文化精华和现代文明成果，在赶超发展的实践中，打造底蕴厚重、特色鲜明的濮阳城市文化。深入开展文明城市创建活动，力争2014年创建成为全国文明城市。

强化公共文化服务。健全现代公共文化服务体系，全面提升公共文化管理服务水平。合理配置文化资源，实施舞台艺术送农民、电影放映下农村等一批文化惠民工程。广泛开展群众性文化活动，丰富群众精神文化生活。实施精品文化工程，繁荣文化艺术创作。加强网络文化建设和管理，净化网络空间。持续开展“扫黄打非”，抵制低俗现象。

大力发展文化产业。注重传承创新，发挥“华夏文明发祥地”、“中国杂技之乡”两大优势，加大对高城遗址、西水坡遗址、瑕丘遗址等文化资源的保护利用，强化宣传推介，组织研讨论证，启动规划编制，力争及早实施，叫响“上古文化看濮阳”。立足“华夏龙都”、“中华帝都”，打造龙文化品牌。加快濮阳国际杂技文化产业园、东北庄原生态杂技文化旅游园区、杂技艺术学校建设，打造面向世界的杂技演艺、教育、旅游、创作、科研、衍生品生产基地，建设名扬全国的杂技之乡。依托黄河自然景观和革命遗址，打造红色旅游、休闲旅游等特色旅游产品。

（九）着力保障改善民生

继续加大民生投入，着力实施好“十项重点民生工程”，切实解决涉及人民群众切身利益的实际问题，让人民群众过上更好生活。

完善就业和社会保障体系。实施就业促进计划，优化就业创业环境，加大就业困难群体帮扶力度，重点做好高校毕业生、农村转移劳动力、下岗失业人员就业工作，城镇新增就业7万人。强化社会保障，提高企业退休人员基本养老金，城镇居民医保和新农合人均财政补助标准提高到320元。完成110万张社会保障卡发放，实现医疗保险省内异地就医即时结算。推行城乡居民大病保险。开工建设各类保障性住房2.5万套，竣工9757套。加快解决滩区安全饮水问题。完善社会养老服务体系，对80岁以上老人发放高龄补贴。加快县级社会福利中心建设。抓好“万村千乡市场工程”、“放心早餐工程”、“家政服务工程”，提高便民服务水平。

大力发展教育、卫生、体育等事业。优先发展教育事业，优化城乡学校布局，促进城镇优质教育资源与农村学校共享，努力实现教育公平。加强教育基础设施建设，建设190所标准化中小学，改善20所农村薄弱中小学校办学条件。启动第二期学前教育三年行动计划。加强职业教育示范院校和特色院校建设，推动职业教育规模化、集约化办学。合理布局医疗资源，加强基层医疗基础设施建设，搞好区域医疗联合体试点、社区医生与居民契约服务试点。实施乡村医疗机构标准化建设攻坚行动，改扩建20所乡镇卫生院，新建改建600所以上村卫生室。完成市五院应急病房楼建设，市眼科医院、市精神卫生中心迁建投用，加快市人民医院病房大楼、市三院口腔综合楼等项目建设。深入开展爱国卫生运动。加快公共体育基础设施建设，开展全民健身活动，竞技体育争创佳绩，积极申办省第十三届运动会。

加强和创新社会治理。加强源头治理，重视社会矛盾排查化解，完善社会稳定风险评估机制，加强应急管理体系和防控体系建设。畅通信访渠道，依法按政策解决好信访问题。创新社区管理，健全基层综合服务管理平台和矛盾纠纷调处机制。完善和创新流动人口、特殊人群管理服务机制。做好第八届村委会换届选举工作。加强民主法制建设，开展普法教育，强化依法治理。扎实开展食品安全示范市、县创建活动，推进食品安全溯源监管综合平台建设，提升食品安全保障水平。全面落实安全生产责任制，坚决遏制重特大事故发生。大力整顿和规范市场秩序，严厉打击制售假冒伪劣行为。完善社会治安防控体系，坚决打击违法犯罪活动，确保社会大局和谐稳定，建设平安濮阳。

继续加强新形势下国防教育和国防后备力量建设，支持驻濮解放军、武警部队、预备役部队、民兵和人民防空建设，完善落实拥军优属政策措施，深入开展双拥共建，巩固军政军民团结。支持工会、共青团、妇联等人民团体发挥桥梁纽带作用。做好全国第三次经济普查工作。统筹做好人口计生、统计、发展研究、民族宗教、外事侨务、残疾人、机关事务、公积金管理、气象、地震、史志、档案等工作。

（十）加强生态文明建设

强化节能减排。加强减排工程建设，确保完成省定减排目标。强化污染防治，组织实施“蓝天工程”、“碧水工程”、“乡村清洁工程”，加强工业和城市大气污染综合治理，开展城市河流清洁行动和饮用水源保护，开展土壤环境保护和农村环境综合整治。升级改造4个空气质量监测站。提高资源利用效率，推进企业清洁生产，减少污染排放。加大环境执法力度，严肃查处环境违法行为。高效利用土地资源，做到城市用地节约集约、工业发展集约高效、现代农业集约经营。

大力发展循环经济。做好循环经济发展规划，启动一批循环化改造项目，逐步构建企业内循环和企业之间、产业之间大循环体系，促进资源循环利用和废弃物再生利用。抓好开发区国家循环化改造示范试点建设，启动中水回用、危废集中处置中心、公共管网等项目建设。

加强生态系统建设。加快生态林业体系建设，启动东部防护林带、南部金堤河生态涵养带、西部生态绿化带、北部龙山生态绿化带、晋豫鲁铁路防护林带等生态林系建设。抓好濮范高速、大广高速和干线公路的廊道绿化。植树2000万株以上，完成生态工程造林12万亩，全市林木覆盖率达到24%。抓好黄河湿地、金堤河湿地、绿城路湿地等生态项目建设。推进全域河流综合治理，让水活起来、岸绿起来。

各位代表！濮阳是我们赖以生存的家园，我们必须更加注重生态文明建设，不但要生活得殷实富足，更要让人民群众永享碧水蓝天！

四、努力建设人民群众满意的政府

坚持为民务实清廉，深入开展党的群众路线教育实践活动，持续抓好“一创双优”和“两转两提”，切实解决“四风”突出问题，着力转作风、促发展、惠民生，建设法治政府、创新政府、诚信政府、廉洁政府。

转变政府职能。创新政府管理理念和方式，强化政府公共服务、市场监管、社会管理、环境保护职责，推动政府职能向创造良好发展环境、提供优质服务、维护社会公平正义、提高社会管理水平转变。继续简政放权，再取消压减下放一批行政审批事项，清理前置条件，严格行政审批准入，规范管理审批事项，逐步推行“负面清单”管理。完善公共服务提供机制，加大购买服务力度，调动市场力量发展公共服务业。完善目标考核体系，加强政府绩效管理，提高行政效能。加强公务员队伍建设，提高素质能力，提供优质高效服务，当好人民公仆。

推进依法行政。强化法治意识，严格按照法定权限和程序行使权力、履行职责。完善决策程序，做到依法科学民主决策。实行行政职权目录清单管理，公开权力主体和程序，把行政权力关进制度的笼子里，让权力在阳光下运行。自觉接受人大法律监督、政协民主监督、人民群众社会监督和新闻媒体监督，认真落实人大决议，办理好人大议案、代表建议和政协提案。广泛听取民主党派、工商联、无党派人士和人民团体意见。加强和完善政府内部层级监督，强化监察、审计等专门监督。推行服务型行政执法，加强行政复议和行政执法监督，严格行政执法责任和过错追究。

坚持务实重干。强化宗旨意识，把人民群众满意作为政府工作的根本准则，多做打基础、利长远的事情，不摆花架子，不做表面文章，不搞形象工程。坚持求真务实，注重深入实际，调查研究，倾听群众呼声，察实情，办实事，解难题。坚持真抓实干，早干快干多干，一级干给一级看，一级带着一级干，扎扎实实干好每一项工作。敢于担当，强化问责，做到令行禁止，政令畅通，切实提高执行力。

打造诚信政府。诚信是一个地方最宝贵、最闪光的名片，必须倍加珍惜。要加强诚信体系建设，切实强化诚信教育，以政务诚信带动商务诚信和社会诚信。公务人员要以身作则，重信践诺，带头讲诚信，做诚实人，办诚信事，维护好诚信濮阳形象。

做到清正廉洁。切实加强廉政建设，不断完善惩防体系，全面落实廉政建设责任制，严格遵守廉洁自律各项规定，加大案件查办力度，坚决查处腐败行为，保持反腐败的高压态势。深入贯彻中央八项规定精神和省、市委有关要求，狠刹“四风”，坚决纠正损害群众利益的不正之风。坚持厉行节约，反对铺张浪费，启动公务用车制度改革，确保“三公”经费只减不增，树清风正气，政府过紧日子，群众过好日子。

各位代表！科学发展、赶超发展是我们的神圣使命，人民群众对幸福生活的热切期盼是我们的历史责任。让我们紧密团结在以习近平同志为总书记的党中央周围，在省委、省政府和市委的坚强领导下，凝心聚力，拼搏进取，确保实现“二三五”赶超目标，为加快建设富裕和谐美丽新濮阳而努力奋斗！
